# Who is providing HIV testing services? The profile of lay counsellors providing HIV testing services in Johannesburg, South Africa in the treat-all era

**DOI:** 10.1186/s12913-023-10331-y

**Published:** 2023-12-07

**Authors:** Idah Mokhele, Tembeka Sineke, Marnie Vujovic, Robert A.C. Ruiter, Dorina Onoya

**Affiliations:** 1 Health Economics and Epidemiology Research Office, Faculty of Health Sciences, University of the Witwatersrand, Johannesburg, South Africa; 2 Anova Health Institute, Johannesburg, South Africa; 3 Faculty of Psychology and Neuroscience , Maastricht University, Maastricht, the Netherlands

**Keywords:** Lay HIV counsellors, HIV testing services, Counselling, South Africa

## Abstract

**Background:**

Lay counsellors are critical in sustaining access to HIV testing services (HTS) and psychosocial support for persons living with HIV (PLHIV). We aimed to describe the professional and psychosocial profiles of lay counsellors in primary healthcare (PHC) clinics in Johannesburg, South Africa under the universal-test-and-treat (UTT) policy context.

**Methods:**

We conducted a descriptive analysis of a cross-sectional survey among adult (≥ 18 years) lay counsellors from 20 PHC facilities (2–3/ clinic) in Johannesburg, South Africa. Consenting counsellors were interviewed between June 2018 and March 2019. We report on counsellors’ demographic profiles, training, work experience, and mental and emotional well-being.

**Results:**

Overall, 55 consenting adult (≥ 18 years) lay counsellors (92.7% female, median age 37 years, interquartile range [IQR]: 33–44, and 27.3% HIV diagnosed) were surveyed. Most (85.5%) were Department of Health lay counsellors receiving a volunteer stipend at the time. Overall, 56.4% had been working as counsellors for five years or longer. The majority (87%) had completed the National HIV Testing Services Policy Guidelines-recommended 10-day basic counselling training, but 45.2% had not completed refresher training within the guideline’s required 24 months. Reported operational barriers include lack of designated space for counselling (56.4%), inadequate professional supervision and support (40.7%) and insufficient emotional support (over 56.4%), and 60% were overwhelmed by their workload. A total of 18.2% had major depressive symptoms, and the same proportion scored low for psychological well-being. While most (87.3%) reported moderate job satisfaction, 50.9% actively sought alternative employment.

**Conclusion:**

Despite lay counsellors’ significant role in delivering HIV care in South Africa, there has been minimal investment in their skills development, emotional support, and integration into the formal health workforce. Counsellors’ persisting unmet psychosocial, training, and professional needs could impact their efficacy in the UTT era.

## Introduction

Achieving universal antiretroviral therapy (ART) coverage is intrinsically linked to sustained access to HIV testing services (HTS) as an entry point to HIV prevention, treatment, care, and support. Over the years, the South African government has increased the availability, quality, and uptake of HIV testing services as part of its HIV response. Consequently, the proportion of people who tested for HIV increased by 45% between 2005 and 2017 [1, 2]. Furthermore, by 2020, 5.2 million of the 7.9 million persons living with HIV (PLHIV) were initiated on life-saving, life-long ART [3, 4]. Despite these achievements, declines in new HIV infections remain below the UNAIDS target of a 75% reduction of new infections by 2025 [5–7]. Moreover, nearly 3 million persons with known positive HIV status remain untreated, with persistent threats of attrition from the ART program [8–10]. More remains to be done to fast-track the progress towards universal ART coverage and achieve the expanded 95-95-95 targets to end the HIV epidemic by 2030 [4].

Like many countries in Sub-Saharan Africa with a high HIV burden and a shortage of health professionals, South Africa adopted the World Health Organization’s (WHO) task-shifting strategy to increase capacity in its HIV treatment program by using lower-level cadres of health workers [11–16]. Consequently, HIV lay counsellors are heavily relied upon to deliver HTS in the South African national HIV program. Since implementing the National HIV Testing and Counselling (HTC) campaign in 2010, where lay counsellors were at the forefront of HIV counselling and testing, over 13 million people had tested for HIV by mid-2011 [17]. Additionally, since HIV testing and counselling were task-shifted to lay counsellors, an estimated proportion of adults tested for HIV increased from 47.3% to 2010 to 76.3% in 2019 [18, 19], with ART coverage rising by over 50% during the same period [18, 19].

Strategies to improve ART coverage depend primarily on identifying and successfully linking individuals unaware of their HIV status to ART [20]. The recent adoption of the universal test-and-treat policy, same-day ART initiation, and implementation of the National Adherence Guidelines for Chronic Diseases (HIV, tuberculosis, and non-communicable diseases) centres around the role of lay counsellors [21, 22]. Consequently, lay counsellors are central to South Africa, meeting the first 90 of the UNAIDS target and the ongoing HIV case-finding efforts [13, 14, 23].

Lay counsellors are health workers without tertiary education who provide HIV testing and ART adherence counselling to PLHIV managed at primary health facilities [24]. Historically, lay counsellors were supported through donor-funded non-governmental organizations (NGOs) mandated to support the expansion of the HIV programs of high-burden, low and middle-income countries (LMIC) [25–27]. However, there have been persistent challenges to the optimal integration of lay counsellors in the South African primary health care system [14, 16, 24, 28–31]. As donor funding to NGOs declined in upper-middle-income countries like South Africa, the financial and supervisory responsibility for HTS was delegated to provincial health departments as part of the Expanded Public Works Program (EPWP) [27, 29, 32]. However, there is still no national strategy governing this cadre of health workers’ management, training, scope of practice, and remuneration [14, 33]. Therefore, we need to determine how lay counsellors manage the increased scope of work under the universal and early ART strategy and whether they are adequately equipped and supported to deliver good quality HTS in the current policy context in South Africa.

We aim to describe the demographic characteristics, training background, work experience, current work context, and emotional well-being of lay counsellors providing HIV testing services at primary healthcare clinics in Johannesburg, South Africa. The objective is to identify gaps in HTS service provision in the context of the current universal and early ART policy and propose strategies to increase lay counsellor capacity within the South African public healthcare sector.

## Methods

### Study design, setting and participants

We conducted a descriptive analysis of a cross-sectional survey among adult ( ≥ 18 years) lay counsellors working in PHC facilities in Johannesburg, South Africa. Data analyzed as part of this cross-section study was collected in a baseline survey of a pilot trial that aimed to evaluate the effectiveness of a 12-month motivational interviewing counselling training and support program named: Thusa-Thuso, “*helping you help*” among lay counsellors working in 20 PHC clinics in Johannesburg, South Africa (Pan African Clinical Trial Registry (www.pactr.org) database, trial registration number: PACTR202212796722256). A total of 10 clinics were randomized to receive the training intervention, and an additional 10 continued with the standard practice. All participants provided written informed consent to participate in the study. Informed consent was administered in the participant’s preferred language (English, Sotho or Zulu).

The study included interviewer-administered baseline study interviews using semi-structured study questionnaires available in English, Sotho or Zulu. Baseline interviews were conducted with consenting lay counsellors from all study sites from June 2018 to March 2019. We collected information on lay counsellors’ socio-demographic characteristics, work experience, training background, work context, and emotional and psychosocial well-being.

### Measures

We assessed ART knowledge based on responses to 10 ART knowledge index (scored 1 for a correct answer) and categorized total knowledge scores as “Low” (score < 7) or “Medium to high” (score ≥ 7). Negative attitudes toward PLHIV was assessed using an adapted six-item 4-point Likert scale (1 = strongly agree to 4 = strongly disagree) (Cronbach’s alpha = 0.74) originally developed in South Africa [34]. Examples of questions included, “people who have HIV should be ashamed”, and “a person with HIV must have done something wrong and deserves to be punished”. We categorized mean scores into low (0), medium (1 to 2), or high (≥ 3) negative attitudes.

We collected data on participants’ work history and current work context. This encompassed their current duties, the number of years they worked as lay counsellors and the type of HIV testing they are experienced in providing. Additionally, the population groups they have experience in testing, their working hours per week, the number of clients seen per day, and the average time spent with each client. We also enquired about their current employer (Department of Health, NGO support partners), employment status (full-time worker, part-time worker, or volunteer), and who they considered their primary supervisor.

We used the job satisfaction survey (JSS), a 10-item, four-point scale evaluating participants’ feelings or reactions towards different aspects of their jobs, including receiving recognition for a job well done, a good salary, and feeling secure about their job (Cronbach’s alpha = 0.79) [35, 36]. We categorized mean scores as low (score < 2), medium (score 2 to < 3), or high (≥ 3) job satisfaction. We used an 8-item, five-point scale (1 = never to 5 = always) to assess lay counsellors’ experience of inadequate factors in their work environment. Examples include a lack of designated space for counselling, inadequate materials/equipment for performing their duties and feeling overwhelmed by their workload. We dichotomized final scores as rarely-to-always (2 to 5) or never (1).

We then measured perceived social support (PSS) using an eight-item, four-point scale in which participants indicated their overall level of satisfaction with the support available to them (Cronbach’s alpha = 0.72) [37]. We computed mean scores and categorized them as low (< 2), medium (2 to < 3), or high PSS (≥ 3). Next, we assessed psychological well-being using Ryff’s shortened 18-item, six-point psychological well-being (PWB) scale (Cronbach’s alpha = 0.63) [38]. We categorized mean scores as low (< 3.5), medium (3.5 to < 4.5), or high PSS (≥ 4.5). Lastly, depression was measured using the Centre for Epidemiologic Studies-Depression (CES-D) 10 scale, a 10-item questionnaire with a four-point scale (scores range from 0 to 3) that measures general depressive symptoms experienced up to 7 days prior (Cronbach’s alpha = 0.78) [39, 40]. We categorized mean scores into no depression (CES-D 10 total score < 5), low to medium depression (CES-D 10 total score ≥ 5 and < 12) and major depressive symptoms (CES-D 10 total score ≥ 12) [23, 24].

We developed a household amenities index through factor analysis of participants’ household characteristics (type of toilet facilities, energy used for cooking, housing structure, household density, and food availability) and household assets (television, radio, refrigerator, satellite television, cellular telephone, landline telephone, microwave oven, and personal computer) [25]. The total score for the household wealth index ranged from 0 to 1, with a higher total score reflecting better access to amenities (Cronbach’s alpha = 0.81). A cut-off score of 0.3 or less indicated a “low” amenities score, above 0.3 to 0.67 indicated a “medium” amenities score and a score higher than 0.67 indicated “high” amenities score. Other socio-demographic factors assessed include sex, age, marital status, and type of house they live in.

### Analysis

We used descriptive statistics to summarise lay counsellor demographic characteristics, training background, work experience, current work context, and emotional well-being. Continuous variables were described using medians and interquartile ranges (IQR) where appropriate. Categorical variables are described using frequencies and percentages. Analysis was conducted using STATA version 14 (Stata Corp, College Station, Texas, USA).

### Ethical review

The study was approved by the Human Research Ethics Committee (Medical) of the University of the Witwatersrand (Wits HREC M170579). Accordingly, all personal identifiers, including participants’ and facility names, were removed from the final analytic dataset.

## Results

**Fig. 1:**
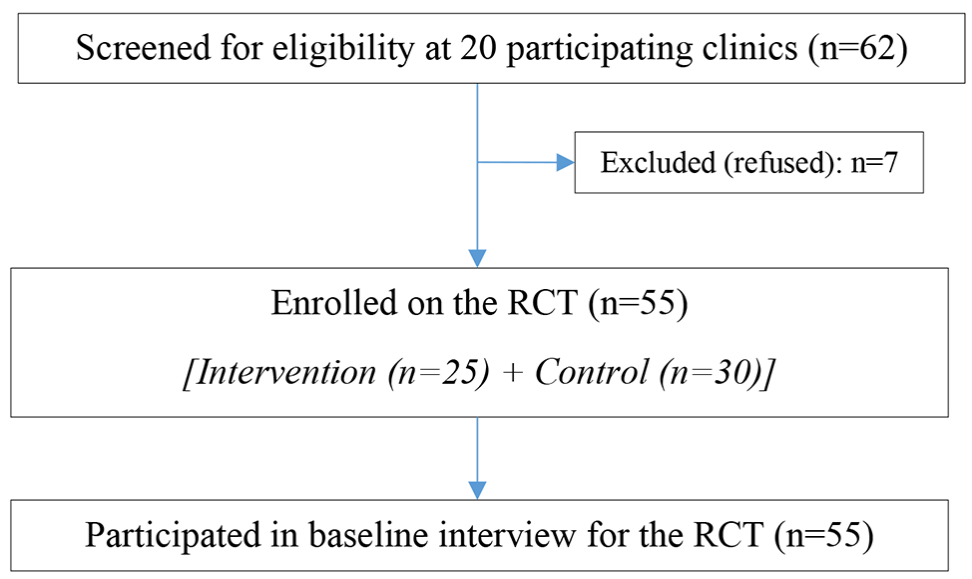
Recruitment and enrolment of study participants in the parent study (RCT)

We approached 20 primary healthcare clinics to participate in the study, and all agreed. However, seven of the 62 lay counsellors at the 20 clinic sites refused to participate (Fig. 1). Participants who refused were all NGO-employed lay counsellors. The main reasons for refusing include being too busy and trying to meet daily targets. Overall, 55 lay counsellors agreed to participate in the study and completed the baseline survey.

### Socio-demographic characteristics

Table 1 reports the socio-demographic characteristics of lay counsellors enrolled in the study stratified by years of experience study participants had been working as lay counsellors. Most lay counsellors were female (92.7%), with a median age at study enrolment of 37 years (IQR: 33.0–44.0). Overall, 56.4% of lay counsellors had been working as counsellors for five years or longer. More experienced (> 5 years) lay counsellors were also older, 40 years (IQR: 38.5–47.0) compared to the less experienced, median age of 35 years (IQR: 35.0–46.0). In addition, a higher proportion of HIV-positive lay counsellors had more than ten years of counselling experience compared to their HIV-negative and non-disclosed counterparts, respectively (40% vs. 15.1% vs. 16.7%).

**Table 1:** Counsellor baseline socio-demographic characteristics as a function of years of experience (5 years or less versus more than 5 years) (n = 55)

	<=5 years	> 5years	Total
	No. (%)	No. (%)	No. (%)
**Sex**			
Female	23 (95.8)	28 (90.3)	51 (92.7)
Male	1 (4.2)	3 (9.7)	4 (7.3)
**Age at study enrolment, years Median (IQR)**	34 (60.5–39.0)	40 (35.0–46.0)	37 (33.0–44.0)
24-29.99	5 (20.8)	2 (6.5)	7 (12.7)
30-34.99	9 (37.5)	5 (16.1)	14 (25.5)
35-39.99	5 (20.8)	7 (22.6)	12 (21.8)
40+	5 (20.8)	17 (54.8)	22 (40.0)
**Marital status**			
Married	6 (25.0)	6 (19.4)	12 (21.8)
In a relationship	13 (54.2)	18 (58.1)	31 (56.4)
Single, no partner	5 (20.8)	7 (22.6)	12 (21.8)
**Lives in**			
Own home/renting	13 (59.1)	24 (77.4)	37 (69.8)
Family/partner/relative’s home	9 (40.9)	7 (22.6)	16 (30.2)
**Type of house**			
House or brick structure in its own separate stand/yard	14 (60.9)	17 (54.8)	31 (57.4)
House/flat/room in someone else’s house or yard	3 (13.0)	8 (25.8)	11 (20.4)
Informal dwelling or shack	6 (26.1)	6 (19.4)	12 (22.2)
**Access to basic necessities (amenities score)**			
Low	2 (10.0)	3 (10.3)	5 (10.2)
Medium	8 (40.0)	17 (58.6)	25 (51.0)
High	10 (50.0)	9 (31.0)	19 (38.8)
**HIV status**			
HIV negative	18 (75.0)	15 (48.4)	33 (60.0)
HIV positive	4 (16.7)	11 (35.5)	15 (27.3)
Declined to disclose	2 (8.3)	5 (16.1)	7 (12.7)
**HIV testing history**			
last HIV test < 3 months ago	17 (70.8)	12 (40.0)	29 (53.7)
last HIV test 6–12 months ago	2 (8.3)	5 (16.7)	7 (13.0)
last HIV test > 12 months ago	5 (20.8)	13 (43.3)	18 (33.3)
**Highest level of education**			
High school	5 (20.8)	8 (25.8)	13 (23.6)
Completed Grade 12	9 (37.5)	10 (32.3)	19 (34.5)
>Grade 12	10 (41.7)	13 (41.9)	23 (41.8)
**English literacy**			
I can read very well	21 (87.5)	26 (83.9)	47 (85.5)
I can read somewhat	3 (12.5)	5 (16.1)	8 (14.5)
**Perceived social support**			
Medium	10 (41.7)	13 (41.9)	23 (41.8)
High	14 (58.3)	18 (58.1)	32 (58.2)
**Psychological well-being**			
Low	3 (12.5)	7 (22.6)	10 (18.2)
Moderate	19 (79.2)	18 (58.1)	37 (67.3)
high	2 (8.3)	6 (19.4)	8 (14.5)
**Depression**			
No depression	14 (58.3)	22 (71.0)	36 (65.5)
low to med depression	2 (8.3)	7 (22.6)	9 (16.4)
Major depression	8 (33.3)	2 (6.5)	10 (18.2)
**Coping support**			
None	17 (70.8)	21 (67.7)	38 (69.1)
Church activities	2 (8.3)	4 (12.9)	6 (10.9)
Sport/Exercise	3 (12.5)	1 (3.2)	4 (7.3)
Activities at work	-	4 (12.9)	4 (7.3)
Other	2 (8.3)	1 (3.2)	3 (5.5)
**Attitude towards PLHIV**			
No negative HIV perceptions	23 (95.8)	26 (83.9)	49 (89.1)
Low negative HIV perceptions	1 (4.2)	5 (16.1)	6 (10.9)

*HIV, human immunodeficiency virus; PLHIV, persons living with HIV; IQR, interquartile range*

A total of (12/55) 21.8% of study participants were married, and 56.4% were in a relationship. Most (69.8%) lived in their own house or rented property, but 22.2% lived in an informal dwelling or shack. A higher proportion of lay counsellors with longer work experience lived in their own homes or were renting (77.4% for > 5 years vs. 59.1% for ≤ 5 years). Overall, 10% of study participants reported low access to basic household amenities compared to 38.8% reporting it as high.

Overall, 27.8% of the lay counsellors reported living with HIV, and 11.1% declined to disclose their HIV status. Among HIV-negative lay counsellors, the majority, 30/33 (90.9%), last tested for HIV within 12 months before study enrolment, with most 26/33 (78.8%) last testing three months before study enrolment. Conversely, only 3/15 of the HIV-positive counsellors were diagnosed in the prior 12 months.

### Psychosocial well-being

Over a third of the lay counsellors reported experiencing depressive symptoms; 16.4% experienced low to medium depression, while 18.2% screened positive for major depressive symptoms. A higher proportion of less experienced counsellors (33.3%) reported experiencing depressive symptoms than their more experienced counterparts (6.5%). Moreover, (18.2%) of study participants also reported low psychological well-being, and over two-thirds had moderate psychological well-being. Most lay counsellors (69.1%) did not participate in activities to help cope with the emotional demands of their work. The few people who participated in debriefing activities either participated in church activities (10.9%), sports and exercise (7.3%), and activities at work (7.3%) to cope.

### Training background and work experience

Although most lay counsellors (85.5%) self-reported high English literacy, 13/55 (23.6%) did not complete high school, and only 41.8% had post-secondary education (Table 2). A quarter (25.8%) of more experienced lay counsellors did not complete high school compared to a fifth (20.8%) among the less experienced.

**Table 2:** Lay Counsellor training background and work experience by the level of education (n = 55)

	<Grade 12 education	>=Grade 12 education	Total
	No (%)	No (%)	No (%)
**English literacy**			
I can read very well	9 (69.2)	38 (90.5)	47 (85.5)
I can read somewhat	4 (30.8)	4 (9.5)	8 (14.5)
**Duration of basic HIV counselling and testing training**			
< 10 days	2 (15.4)	5 (12.2)	7 (13.0)
>=10 days	11 (84.6)	36 (87.8)	47 (87.0)
**Refresher training attendance among those due**			
Yes	6 (60.0)	17 (53.1)	23 (54.8)
No	4 (40.0)	15 (46.9)	19 (45.2)
**Lay counsellor work experience**			
<=5 years	5 (38.5)	19 (45.2)	24 (43.6)
> 5 years	8 (61.5)	23 (54.8)	31 (56.4)
**ART knowledge**			
Medium	2 (15.4)	11 (26.2)	13 (23.6)
High	11 (84.6)	31 (73.8)	42 (76.4)

HIV, human immunodeficiency virus; ART, antiretroviral therapy

All lay counsellors reported receiving basic HIV counselling and testing training. However, 13.0% attended a training program of fewer than the guideline-recommended ten days; all of these were participants with five years or less of work experience as lay counsellors. Furthermore, among those who first attended training 24 months or more before study enrolment (42/55), 45.2% (19/42) had not attended the required refresher training. Over 50% (13/19) of those eligible who did not participate in refresher training were less experienced counsellors (recent entrants into the profession).

**Fig. 2:**
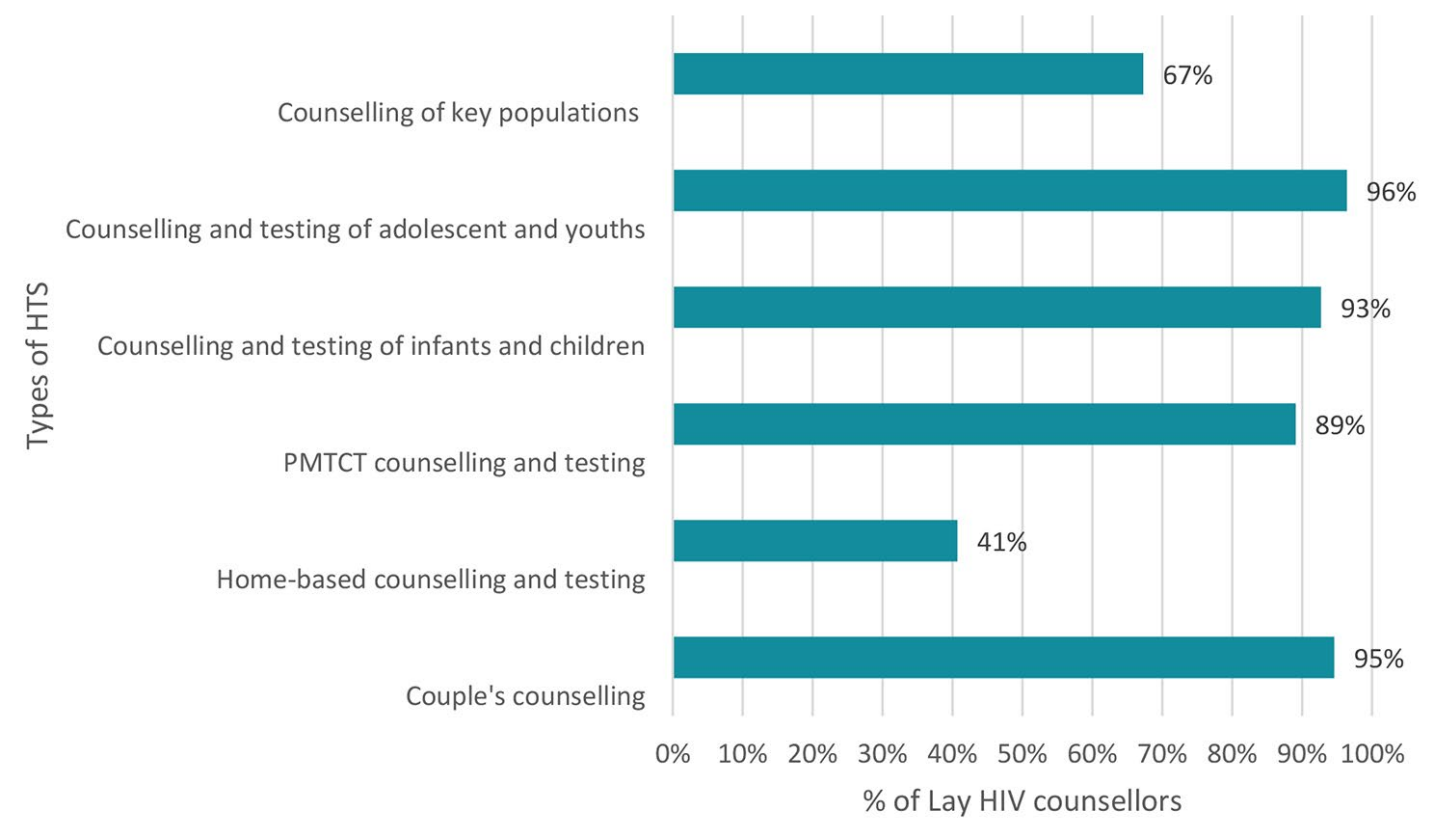
Types of HIV counselling and testing lay counsellors are experienced in providing (n = 55)

Most of the lay counsellors reported some experience with couples counselling (95%), testing adolescents and youth (96%), infants and children (93%), and prevention of mother-to-child transmission (PMTCT) testing among pregnant mothers (89%) (Fig. 2). However, a lower proportion (67.0%) of lay counsellors were experienced in providing HIV testing services to key populations and conducting home-based HIV testing.

ART knowledge was high, with 76.4% of the lay counsellors scoring high. However, almost 10% of the counsellor did not know HIV could be prevented by ART (post-exposure prophylaxis) after rape, and 5% believed that HIV could be cured by ART.

### Work conditions and workload

Most (85.5%) of the study participants were Department of Health lay counsellors receiving a volunteer stipend at the time; 14.5% were NGO lay counsellors deployed to their respective primary healthcare clinics (Table 3). Overall, only 37.5% (3/8) of the NGO lay counsellors had five years or more experience as a lay counsellor compared to 60% of Department of Health lay counsellors.

**Table 3:** Lay counsellor work conditions and workload by type of employer at study enrolment (n = 55)

	Department of Health	NGO - Support partners	Total
	No (%)	No (%)	No (%)
**Number of clients daily, Median (IQR)**	10 (8–11)	11 (8–12)	10 (8–12)
**Average time spent with each client**			
15–30 min	13 (27.7)	1 (12.5)	14 (25.5)
> 30 min	34 (72.4)	7 (87.5)	41 (74.6)
**Main supervisor**			
Facility Manager	33 (73.3)	1 (12.5)	34 (64.2)
NGO-partner mentor/supervisor	2 (4.4)	6 (75.0)	8 (15.1)
DOH mentor/supervisor	10 (22.2)	1 (12.5)	11 (20.8)
**Job satisfaction**			
High	2 (4.3)	-	2 (3.6)
Moderate	42 (89.4)	6 (75.0)	48 (87.3)
Low	3 (6.4)	27 (25.0)	5 (9.1)
**Intention to leave current job**			
low	10 (21.3)	1 (12.5)	11 (20.0)
Med	11 (23.4)	5 (62.5)	16 (29.1)
High	26 (55.3)	2 (25.0)	28 (50.9)

HIV, human immunodeficiency virus; DOH, department of health; NGO, non-governmental organization

Regarding workload, counsellors reported counselling and testing a median of 10 clients (IQR:8–16) daily. Most (74.6%) reported spending more than 30 min with each client on average. In contrast, a quarter reported spending less than 30 min going through the entire HIV counselling and testing process (pre-test counselling, HIV testing and post-test counselling). The majority (87.3%) of the lay counsellors were moderately satisfied with their job, while 9.1% had high job satisfaction. However, 50.91% had high turnover intentions, and 29.1% had moderate intentions to leave their current job.

**Fig. 3:**
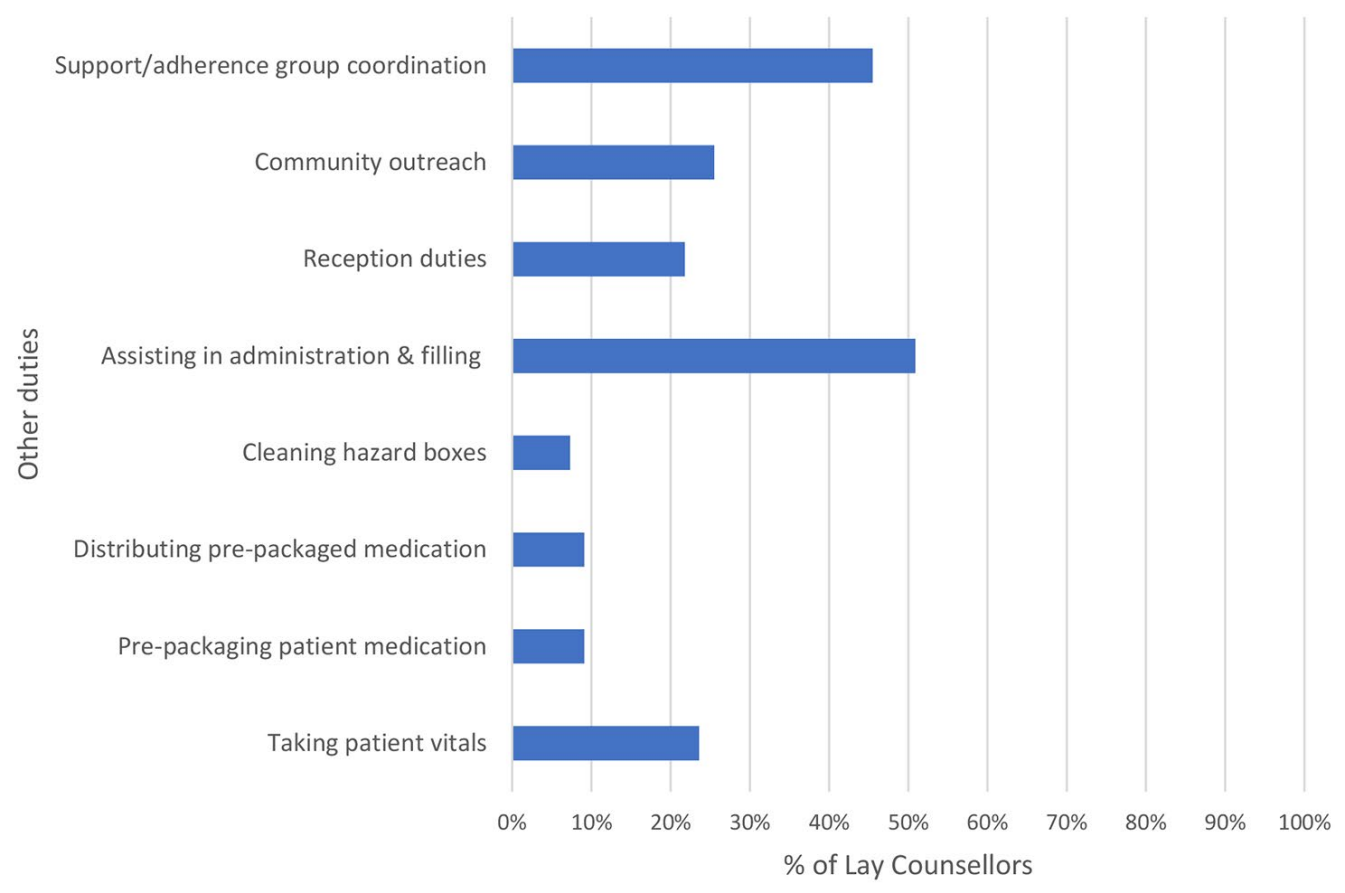
Other duties performed by lay counsellors (n = 55)

Figure 3 details other duties that counsellors reported performing at their facilities. In addition to HIV counselling and testing, adherence counselling, and health education talks, some coordinated support/adherence groups (45.5%) and performed administration duties, including filing patient files (50.9%). Over a fifth of the counsellors reported taking patient vitals and performing reception duties, while 25% also conducted community outreach. A few (less than 10%) reported assisting with taking vitals, pre-packaging and distributing medication, and cleaning hazard boxes.

**Fig. 4:**
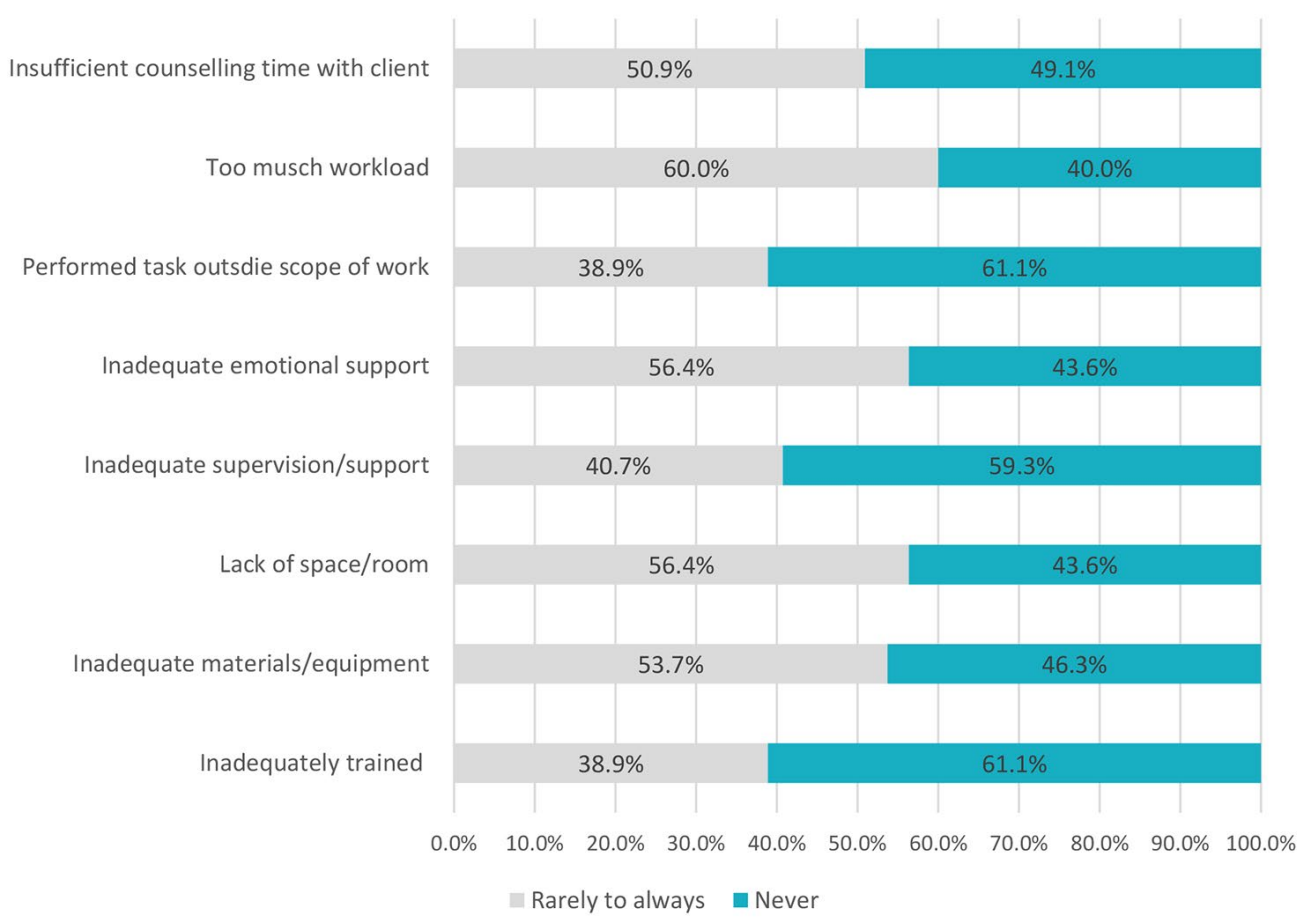
Lay counsellors experience of inadequate factors in their work environment (n = 55)

When asked about challenges related to their work environment, participants reported several operational barriers, including lack of designated space for counselling (56.4%), inadequate materials/equipment for performing their duties (53.7%), and performing tasks outside their scope of work (38.9%) (Fig. 4). Lay counsellors also reported receiving inadequate supervision and support (40.7%) and inadequate emotional support (56.4%). Additionally, over a third reported feeling inadequately trained, and 60.0% felt overwhelmed by their workload. Lastly, a total of 50.9% said that they felt they spent insufficient time counselling clients.

## Discussion

We describe lay counsellors providing HTS in the PHC setting in Johannesburg, South Africa, in the era of universal HIV treatment access. Lay counsellors enrolled in our study primarily comprised female lay counsellors, reflecting the prevailing pattern in South Africa [27, 30, 41]. This gender imbalance can present a limitation when it comes to expanding services to male clients who are less likely to know their HIV status and have suboptimal HIV treatment and prevention coverage [2, 4, 42]. Overall, lay counsellors faced operational barriers in their workplace, including a lack of designated space for counselling, insufficient supervision and support, and emotional support, and a notable 60% felt overwhelmed by their workload. Furthermore, our findings indicate that lay counsellors continue to perform auxiliary duties alongside their primary function of providing HTS. This, coupled with their reported heavy workload, places them at risk of burnout, potentially resulting in a decline in the quality of service provision. Lay counsellors have faced similar challenges in the pre-UTT era since the start of the HTC campaign in 2010 [16, 29, 30]. These challenges indicate continued difficulties clarifying and streamlining their workplace roles and responsibilities and providing adequate supervision and support.

Almost a quarter of the study participants had not completed high school; this is lower than 40–50% reported in previous cohorts evaluated in the pre-UTT era [30, 43]. A grade 12 education is one of the minimum requirements for lay counsellors, as stated in the HTS and Adherence guidelines [21, 23, 44]. Therefore, it may hinder future integration into the health workforce. Moreover, those with low education levels may find it difficult to comprehend training curriculums and may require training approaches that simplify complex concepts, preferably provided in their vernacular.

Although there were gaps in formal education, the current cohort had universal participation in basic HIV counselling and testing training, a considerable improvement from the pre-UTT era when basic training was limited or absent [29, 30, 45]. However, we found training adequacy lower among less experienced counsellors who were also younger. This may suggest a shift in lay counsellors’ training and support landscape over time (less for new entrants). More experienced lay counsellors who developed from HIV peer support in the HIV epidemic’s early years were likely exposed to more training and support opportunities to meet changing policy contexts when donor funding was still plentiful as the national program matured and task-shifting was implemented [13, 33, 46].

Persistent disparities in training and skills among lay counsellors in the UTT era limit the national program’s capacity to achieve universal ART access and end the HIV epidemic by 2030. Observed training gaps highlight the need for lay counsellors to be adequately trained based on current policies and work context supported through quality assurance strategies such as mentoring and routine skill assessments and feedback. In addition, even with high ART knowledge, some study participants were unaware of the preventative properties of ART after rape (post-exposure prophylaxis), and some believed HIV could be cured by ART. Therefore, future training should also address lay counsellor knowledge gaps as scientific advances in HIV and ART continue, including the benefits of early ART and treatment as prevention. Furthermore, existing differences in counsellor profiles may present an opportunity for those with extensive experience and training to be upskilled to become more specialized lay counsellors providing support or mentoring to others. This strategy can also help address the lack of career pathing previously identified in this cadre [28, 29].

Job satisfaction was moderate; a previous pre-UTT cohort had higher job satisfaction [29], which may be indicative of policy changes that may have added complexities to their job with no improvements in the work environment. Additionally, 50% of lay counsellors actively sought alternative employment, with more than twice as many Department of Health lay counsellors having high turnover intentions. These results suggest persistent differences in employment conditions between NGO counsellors and Department of Health lay counsellors, with NGO counsellors earning higher salaries and having clearer reporting lines and supervision through their organizations [14, 33]. Despite these challenges, more counsellors seem confident in their core counselling and testing skills of adolescents, couples, infants and children, whom previous cohorts self-reported having difficulty serving [16, 29, 30]. However, they remain less confident in counselling and testing members of key populations [16, 29, 30].

Almost a fifth of lay counsellors reported experiencing major depressive symptoms; the same proportion experienced low psychosocial well-being. Previous studies have shown below-average emotional well-being and high levels of emotional exhaustion, job stress, and depression among lay counsellors in South Africa [28, 29, 43]. In addition, very few study participants participated in debriefing activities to help them cope with their work’s emotional burdens. Accordingly, there is a very high risk of burnout and an inability to maintain interpersonal sensitivity, which has been previously highlighted [43, 47, 48]. Depressive symptoms and low psychological well-being were more prevalent in those with less counselling experience, possibly due to fewer opportunities to develop coping skills and resilience through differential access to work support over the years. Counsellor training programs should incorporate self-care and debriefing elements to support counsellors in coping. Moreover, ongoing support through workplace debriefing and emotional support programs is also essential to their well-being.

### Limitations

We conducted structured interviews with all counsellors who consented at 20 facilities in the Johannesburg health district. We believe this provides depth and breadth of information into the lay counsellor context in South Africa. However, the study results are limited by the geographic location of participating PHC clinics. Firstly, we only include clinics from the Johannesburg Health District in South Africa. Additionally, the clinics were from urban settings, but there was some diversity in terms of formal versus informal urban settings where facilities served communities residing in informal settlements.

Data collection for the study was conducted before lay counsellors in the district were formally contracted to be Department of Health workers. Additionally, NGO support changed in the district after study enrolment, which saw NGO counsellors either retrenched or moved to other projects. As a result, perspectives regarding their work and environment may have changed.

## Conclusion

Despite the significant role of lay counsellors in expanding access to HIV care in South Africa, little has been done to invest in their ongoing training, emotional support, and integration into the formal health workforce. Counsellors’ persisting unmet psychosocial, training, and professional needs could impact their efficacy in the UTT era.

## Data Availability

The datasets generated and/or zanalyzed during the current study are available from the Health Economics and Epidemiology Research Office for researchers who meet the criteria for access to confidential data and with permission from the owners of the data. Contact the organization at information@heroza.org for additional information regarding data access.

## List of abbreviations

HTS: HIV testing services
PLHIV: Persons living with HIV
PHC: Primary Healthcare clinics
UTT: Universal-test-and-treat
IQR: Interquartile range
HIV: Human Immunodeficiency virus
DOH: Department of Health
NGO: Non-governmental organizations
ART: Antiretroviral therapy
UNAIDS: Joint United Nations Program on HIV/AIDS
WHO: World Health Organisation
LMIC: Low and middle income countries
EPWP: Expanded Public Works Program
JSS: Job satisfaction survey
PSS: Perceived social support
PWB: Psychological well-being
CES-D: Centre for Epidemiologic Studies-Depression
IQR: Interquartile range
Wits: University of the Witwatersrand
HREC: Human research ethics committee
RCT: Randomised control trial
PMTCT: Prevention of mother-to-child transmission
HTC: HIV testing and counselling
